# CRISPR-Cas9 off-target effects in urothelial gene therapy: the overlooked risk of P53 mutational signatures in bladder tissue

**DOI:** 10.1097/MS9.0000000000005079

**Published:** 2026-04-28

**Authors:** Syeda Tamkeen Zahra, Azan Ahmed, Vareesha Batool, Abdul Haseeb Hasan

**Affiliations:** a Department of Medicine, Khyber Medical College, Peshawar, Pakistan; b Department of Medicine, King Edward Medical University, Lahore, Pakistan; c Department of Research, Oli Health Magazine Organization, Research and Education, Kigali, Rwanda; d Department of Internal Medicine, Sentara Northern Virginia Medical Center, VA, USA

*To the Editor,*

Interstitial cystitis (IC), aka bladder pain syndrome (BPS), is a chronic medical condition manifested as pelvic pain, urinary urgency, and frequency in the absence of identifiable infection or structural pathology. It has multifactorial etiologies like immune dysfunction, sensory hyperactivation, and impaired urothelial repair mechanisms. Given its uncertain pathophysiology, the patients of IC/BPS experience refractory symptoms despite conventional management due to a lack of reliable curative therapies, underscoring the need for novel molecular treatments like gene therapy that target underlying mechanisms rather than solely alleviating symptoms[1].

The therapeutic use of gene therapy for IC/BPS, particularly through intravesical delivery, holds strong potential, enabling localized exposure of the bladder urothelium. CRISPR-Cas9-based genome editing, for instance, allows precise, sequence-specific modification of target genes and has demonstrated feasibility through intravesical delivery approaches targeting urothelial tissues, highlighting its potential to influence inflammatory and regenerative pathways implicated in urothelial disorders[2].

The urothelium is a rapidly regenerative tissue with high cellular turnover. While this feature is often considered beneficial for tissue repair, it may also obscure the early detection of low-frequency off-target mutations. *TP53*-mutant cells are rare and arise from unintended Cas9 cleavage activity, which could be temporarily diluted by shedding, yet they may persist within basal or progenitor cell populations. The fact that *TP53* mutations are a defining characteristic of early events in urothelial carcinogenesis, even low-frequency off-target events, carries clinically meaningful long-term risks that are not detected by short-term safety studies[3].

CRISPR–Cas9–induced double-strand breaks induce p53-mediated DNA damage response pathways. Preclinical studies show that genome editing can exert selection pressure on cells with disrupted p53 function, leading to their survival and proliferation. In the setting of bladder epithelium, such selection pressure results in the silent accumulation of TP53-deficient cells without prompt histologic abnormalities or clinical manifestation, thereby delaying recognition of genomic damage. The current discussions mainly emphasize delivery efficiency, local toxicity, and short-term inflammatory responses. However, reduced attention has been paid to potential off-target genomic interactions, especially those involving the TP53 tumor suppressor gene, despite growing evidence that unintended Cas9 activity remains a biologically relevant issue[4].

Despite advancements with high-fidelity Cas9 variants that reduce the incidence of off-target activity, highly sensitive genome-wide detection techniques for identifying rare insertions, deletions, and other structural genomic alterations are still necessary. Notably, the cumulative mutational burden across multiple off-target loci may remain clinically significant, specifically after repeated high-dose intravesical administration of gene-editing platforms. In response to these concerns several preventive and monitoring strategies have been recommended to mitigate the risk of urothelial carcinogenesis linked to CRISPR-based therapies. The clinical application of intravesical CRISPR therapies should also include strategies for longitudinal genomic surveillance. These include off-target mapping in preclinical models by techniques such as CIRCLE-seq, molecular monitoring of exfoliated urothelial cells, extended post-treatment follow-up, and patient counseling about the potential late genomic toxicity[5].

As CRISPR-based therapies emerge as the mainstream therapeutic approach towards the management of non-malignant bladder disease[6], rigorous off-target mitigation strategies and sustained genomic surveillance should not be viewed as afterthoughts but as an integral component of these treatments. A cautionary and surveillance-driven strategy will play a crucial role in balancing therapeutic innovation with long-term patient safety and public trust.

In conclusion, intravesical CRISPR-Cas9 is a promising therapeutic avenue for IC/BPS. However, concerns regarding persistent off-target genomic alterations, particularly in TP53, warrant scrutiny. Given the regenerative nature of the urothelium, early detection may be challenging, reinforcing the need for robust genomic surveillance and long-term clinical monitoring.

## Data Availability

Data sharing not applicable to this article as no datasets were generated or analyzed during the current study
